# Comparative Predictive Performance of EuroSCORE II and the STS Score for Postoperative Cardiopulmonary Complications in Older Adults Undergoing Cardiac Surgery

**DOI:** 10.3390/jcm15135128

**Published:** 2026-07-01

**Authors:** Mantana Saetang, Thitikan Kunapaisal, Sirinporn Limvatanalert, Arinda Sontarapornpol, Wirat Wasinwong, Pongsanae Duangpakdee, Ploychanok Khunpanich, Patrapon Packawatchai

**Affiliations:** 1 Department of Anesthesiology, Faculty of Medicine, Prince of Songkla University, Songkhla 90110, Thailand; mantana.s@psu.ac.th (M.S.); sirinporn.t@psu.ac.th (S.L.); arinda.s@psu.ac.th (A.S.); wirat.w@psu.ac.th (W.W.); ploychanok.k@psu.ac.th (P.K.); patraporn.p@psu.ac.th (P.P.); 2 Department of Surgery, Faculty of Medicine, Prince of Songkla University, Songkhla 90110, Thailand; pongsanae.d@psu.ac.th

**Keywords:** EuroSCORE II, STS score, cardiac complications, pulmonary complications, older patients, cardiac surgery

## Abstract

**Background/Objectives:** Accurate preoperative risk stratification is critical in older adults undergoing cardiac surgery, particularly for predicting postoperative cardiopulmonary complications (PCPCs). Although EuroSCORE II and the Society of Thoracic Surgeons (STS) score are widely used to estimate mortality risk, their ability to predict morbidity in older adults remains unclear. This study compared the performance of EuroSCORE II and the STS score in predicting PCPCs after on-pump cardiac surgery. **Methods:** This retrospective cohort study included patients aged ≥ 60 years who underwent elective on-pump cardiac surgery at Songklanagarind Hospital between January 2017 and December 2022. PCPCs included prolonged mechanical ventilation (>48 h), reintubation within 72 h, pneumonia, respiratory failure, new-onset atrial fibrillation, or low cardiac output requiring inotropic or mechanical support. Predictive performance was evaluated using receiver operating characteristic (ROC) curve analysis, logistic regression, calibration analysis, and overall prediction accuracy measures. **Results:** Among 638 patients, 273 (42.8%) developed PCPCs. Both scores were significantly associated with PCPCs. EuroSCORE II showed modest discrimination (area under the curve [AUC]: 0.581; 95% CI: 0.536–0.626); in contrast, the STS Score performed better (AUC: 0.625; 95% CI: 0.580–0.669; *p* = 0.017). In logistic regression, EuroSCORE II (odds ratio [OR]: 1.06; 95% CI: 1.01–1.10; *p* = 0.006) and the STS Score (OR: 1.05; 95% CI: 1.03–1.07; *p* < 0.001) were independently associated with PCPCs. Both models demonstrated acceptable calibration, although STS score showed better overall predictive performance than EuroSCORE II. **Conclusions:** EuroSCORE II and the STS score were both associated with postoperative cardiopulmonary complications but demonstrated only modest discriminative ability. Although the STS score performed significantly better than EuroSCORE II, neither model achieved sufficient accuracy for clinically meaningful individualized risk prediction.

## 1. Introduction

Cardiac surgery in older adults presents significant challenges because of age-related physiological decline, multiple comorbidities, and an increased risk of postoperative complications [1]. Among these, cardiorespiratory complications including respiratory failure, prolonged mechanical ventilation, arrhythmias, and hemodynamic instability remain major contributors to morbidity, prolonged intensive care unit stays, and increased healthcare burden [2,3]. Accurate preoperative risk stratification is therefore essential to optimize perioperative care, guide resource allocation, and improve patient outcomes.

Over time, various risk assessment models have been developed to predict postoperative complications and mortality [4,5]. Among them, the European System for Cardiac Operative Risk Evaluation II (EuroSCORE II) and the Society of Thoracic Surgeons (STS) score are widely used for preoperative risk assessment in cardiac surgery [6,7,8]. However, these models were originally designed to predict operative mortality, and their ability to predict postoperative cardiorespiratory complications in older adults remains uncertain.

EuroSCORE II was introduced as an update to the original EuroSCORE to address concerns about risk overestimation, particularly in lower-risk populations [9]. The model incorporates patient demographics, comorbidities, and procedural variables to estimate 30-day mortality after cardiac surgery. Although several studies have validated its performance for mortality prediction [9,10,11], its ability to predict nonfatal outcomes, such as respiratory and cardiac complications, has been questioned. Although EuroSCORE II performs well for operative mortality, its accuracy for postoperative morbidity appears limited.

The STS score is a validated risk prediction model derived from the STS National Adult Cardiac Surgery Database [12,13]. It incorporates age, sex, comorbidities (for example, hypertension, peripheral arterial disease, cerebrovascular disease, diabetes, and lung disease), and preoperative clinical status (for example, cardiogenic shock and heart failure). Unlike EuroSCORE II, the STS model provides procedure-specific risk estimates for individual complications [14,15,16], which may make it more suitable for predicting cardiorespiratory morbidity in older adults undergoing cardiac surgery.

Despite the widespread use of EuroSCORE II and the STS score, their comparative performance in predicting cardiorespiratory complications in older adults undergoing on-pump cardiac surgery has not been fully established. Most existing studies have focused primarily on mortality rather than morbidity [9,13,17], leaving an important gap in understanding how well these tools predict clinically significant nonfatal complications.

Therefore, this study aimed to directly compare the predictive performance of EuroSCORE II and the STS score for postoperative cardiorespiratory complications in older adults undergoing on-pump cardiac surgery. Identifying the model with better discriminatory ability may help refine preoperative risk assessment and improve perioperative care strategies for this high-risk population.

## 2. Materials and Methods

### 2.1. Study Design and Setting

This retrospective cohort study was conducted at Songklanagarind Hospital, a tertiary care center in southern Thailand. Data were obtained from the REC.66-215-8-1 study database, which included older adults undergoing cardiac surgery between January 2017 and December 2022. This study protocol was reviewed and approved by the Institutional Ethics Committee of the Faculty of Medicine, Prince of Songkla University, Songkhla, Thailand (REC.68-254-8-1) on 12 July 2025, and a waiver of informed consent was granted.

### 2.2. Study Population

Patients aged ≥ 60 years who underwent elective on-pump cardiac surgery were included. Eligible procedures consisted of coronary artery bypass grafting (CABG), valvular surgery, and combined CABG with valvular surgery. Patients were excluded if they underwent emergency surgery, received off-pump procedures, or had incomplete clinical or outcome data.

### 2.3. Data Collection

Clinical data were extracted from the REC.66-215-8-1 database. Collected variables included demographic characteristics (age, sex, body mass index [BMI]), baseline comorbidities (for example, diabetes mellitus, hypertension, chronic kidney disease, and chronic obstructive pulmonary disease), and cardiac function measures (left ventricular ejection fraction).

Operative variables, including surgery type, cardiopulmonary bypass (CPB) time, and aortic cross-clamp (AXC) time, were also recorded. Preoperative risk stratification scores, specifically EuroSCORE II and the STS score, were obtained. Postoperative outcomes were subsequently collected for analysis.

### 2.4. Risk Score Assessment

Preoperative risk was assessed using two validated models:

- **EuroScore II** [18]: A cardiac risk model for predicting mortality after cardiac surgery. The new set of risk factors consists of patient-, cardiac-, and operation-related factors (http://www.euroscore.org).
- **STS score** [19]: A risk model based on age, sex, comorbidities (for example, hypertension, peripheral arterial disease, cerebrovascular disease, diabetes, and lung disease), and preoperative clinical status (for example, cardiogenic shock and heart failure). The STS score used in this study was the Predicted Risk of Morbidity or Mortality (PROMM), calculated using the official STS Adult Cardiac Surgical Risk Calculator (http://riskcalc.sts.org/stswebriskcalc/, accessed on 15 June 2026).

Both scores were calculated according to standard validated protocols based on preoperative clinical variables.

### 2.5. Outcomes

The primary objective of this study was to compare the predictive performance of EuroSCORE II and the STS score in assessing the risk of cardiorespiratory complications in older adults (aged ≥ 60 years) undergoing on-pump cardiac surgery, including CABG, valvular surgery, and combined CABG and valvular surgery.

### 2.6. Outcome Definitions

The primary outcome was postoperative cardiopulmonary complications (PCPCs), defined as the occurrence of one or more clinically relevant adverse events. These events included prolonged mechanical ventilation (>48 h), reintubation within 72 h of extubation, postoperative pneumonia, respiratory failure, new-onset atrial fibrillation, and low cardiac output syndrome requiring inotropic or mechanical circulatory support. Patients who experienced one or more of these events were classified as having a postoperative cardiopulmonary complication (PCPC). For the composite outcome analysis, each patient contributed only once regardless of the number of individual complications experienced.

### 2.7. Sample Size

The sample size was determined based on the primary objective of comparing the predictive performance of EuroSCORE II and the STS score. The estimation was guided by previously published data. Saetang et al. [20] reported that EuroSCORE II predicted postoperative pulmonary complications with an area under the curve (AUC) of 0.631. Owing to limited evidence regarding the predictive ability of the STS Score for cardiopulmonary complications, the AUC estimate for the STS score was derived from the study by Hassan et al. [14], who reported an AUC of 0.841 for postoperative stroke prediction.

Using a two-sided significance level (α = 0.05) and 80% statistical power, the required sample size was estimated to be 286 patients per group after applying a continuity correction.

$$n_{1}={\left[\frac{z_{1-\frac{\alpha }{2}}\sqrt{\bar{p}\bar{q}\left(1+\frac{1}{r}\right)}+z_{1-\beta }\sqrt{p_{1}q_{1}+\frac{p_{2}q_{2}}{r}}}{\Delta }\right]}^{2}$$

$$r=\frac{n_{2}}{n_{1}},\;q_{1}=1-p_{1},\;q_{2}=1-p_{2}$$

$$\bar{p}=\frac{p_{1}+p_{2}r}{1+r},\;\bar{q}=1-\bar{p}$$

This study used an existing retrospective dataset of 638 older adults, which exceeded the minimum required sample size and was therefore considered adequate.

### 2.8. Statistical Analysis

Continuous variables are presented as mean ± standard deviation (SD) or median (interquartile range [IQR]), as appropriate, and categorical variables as frequencies and percentages. Comparisons between patients with and without PCPCs were performed using the independent *t*-test or Mann–Whitney U test for continuous variables and the chi-square test or Fisher’s exact test for categorical variables, as appropriate.

The associations between risk scores and PCPCs were evaluated using univariable logistic regression analysis and are reported as odds ratios (ORs) with 95% confidence intervals (CIs). The predictive performance of EuroSCORE II and the STS score was assessed using receiver operating characteristic (ROC) curve analysis and the area under the curve (AUC) with 95% CIs. Differences between AUCs were compared using the DeLong test. Optimal cutoff values were determined using the Youden index.

Model calibration was assessed using the Hosmer–Lemeshow goodness-of-fit test. Overall predictive performance was further evaluated using the Brier score, mean absolute error (MAE), and mean squared error (MSE), with lower values indicating better predictive accuracy and calibration. All statistical analyses were performed using R version 4.5.1 (R Foundation for Statistical Computing, Vienna, Austria). A two-sided *p*-value < 0.05 was considered statistically significant.

## 3. Results

**Figure 1:**
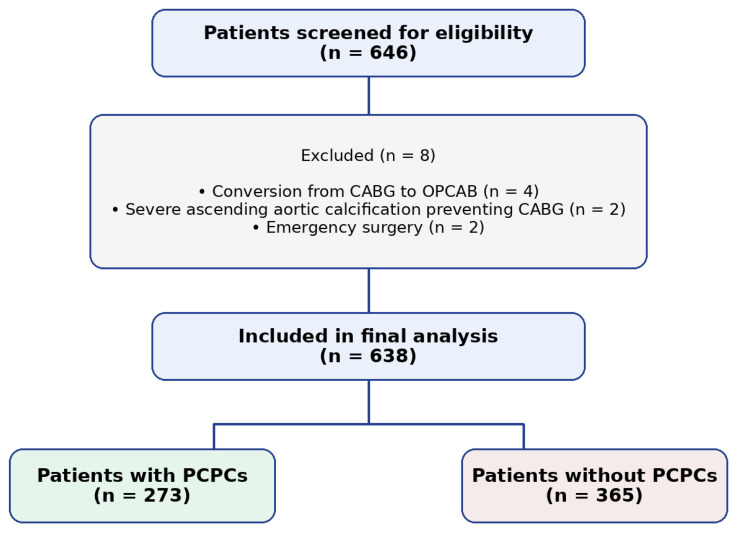
Flow diagram of patient screening, exclusion, and inclusion in the final analysis.

A total of 638 older adults were included in the analysis (Figure 1), of whom 273 (42.8%) developed PCPCs. These complications included respiratory events—such as prolonged mechanical ventilation, atelectasis, and pneumonia—and cardiac events, including postoperative arrhythmias and low cardiac output syndrome requiring intra-aortic balloon pump (Table 1).

### 3.1. Baseline Characteristics

There were no significant differences in age between patients with and without PCPCs (median age: 69 [64–74] vs. 68 [63–73] years; *p* = 0.342). Sex distribution and BMI were also comparable between groups. However, patients who developed PCPCs had significantly lower body weight (60 [53–67] kg vs. 62 [54–70] kg; *p* = 0.027) (Table 2). A higher prevalence of congestive heart failure was observed in patients with PCPCs compared with those without complications (47.3% vs. 29.0%; *p* < 0.001). Similarly, New York Heart Association (NYHA) functional class III–IV was more common in the PCPC group (37.0% vs. 23.8%; *p* < 0.001). Preoperative anemia was also more frequent among patients who developed PCPCs (63.7% vs. 51.2%; *p* = 0.002) (Table 2).

### 3.2. Preoperative Risk Scores

Both risk scores were significantly higher in patients who developed PCPCs. The median EuroSCORE II was 2.3 (1.4–4.0) in the complication group compared with 1.8 (1.2–3.0) in the non-complication group (*p* < 0.001). Similarly, the median STS score was 13.4 (8.1–21.4) vs. 9.8 (6.7–14.5), respectively (*p* < 0.001). Frailty, assessed using the modified Frailty Index-11, also differed significantly between groups (*p* = 0.002) (Table 2).

### 3.3. Operative Characteristics

Patients who developed PCPCs had significantly longer operative durations. The median operative time was 330 (285–390) min in the complications group compared with 310 (270–375) min in the non-complications group (*p* = 0.007). CPB time and AXC time were also significantly longer in patients who developed PCPCs (*p* = 0.019 and *p* = 0.020, respectively). In addition, patients with PCPCs required significantly greater transfusion volumes. The median packed red cell transfusion volume was higher in the complications group (481 [277.5–610.5] mL) than in the non-complications group (319 [238–505] mL) (*p* < 0.001). Fresh frozen plasma and platelet concentrate transfusion volumes were likewise significantly greater among patients with PCPCs (*p* = 0.019 and *p* = 0.027, respectively) (Table 2).

### 3.4. Predictive Performance of Risk Scores

**Figure 2:**
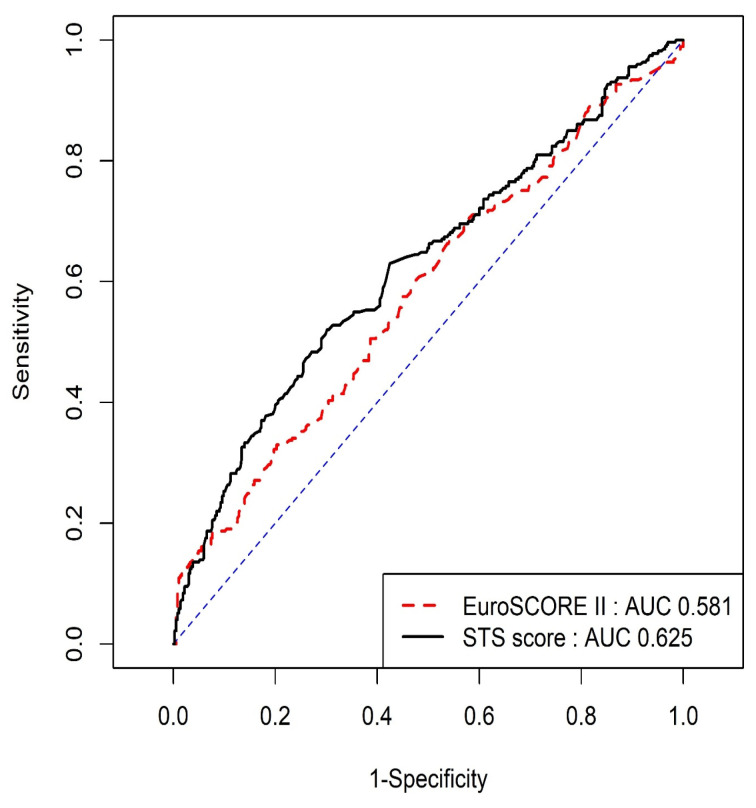
ROC curves of EuroSCORE II and the STS score in PCPCs. ROC, receiver operating characteristics; EuroSCORE II, The European System for Cardiac Operative Risk Evaluation II; STS, Society of Thoracic Surgeons; PCPCs, postoperative cardiopulmonary complications.

The discriminative abilities of the EuroSCORE II and the STS score for predicting PCPCs were evaluated using receiver operating characteristic (ROC) curve analysis. EuroSCORE II demonstrated modest predictive performance, with an AUC of 0.581 (95% confidence interval [CI]: 0.536–0.626). In comparison, the STS score showed superior discrimination, with an AUC of 0.625 (95% CI: 0.580–0.669) (Figure 2). Direct comparison of the ROC curves indicated that the STS Score provided significantly better predictive accuracy than EuroSCORE II (*p* = 0.017).

Model calibration and overall predictive performance were further evaluated (Table 3). The STS score demonstrated better overall predictive accuracy than EuroSCORE II, with a lower Brier score (0.231 vs. 0.240), lower calibration mean absolute error (MAE: 0.017 vs. 0.030), and lower calibration mean squared error (MSE: 0.00043 vs. 0.00405). Calibration assessment using the Hosmer–Lemeshow goodness-of-fit test showed no evidence of poor calibration for either model (EuroSCORE II: χ^2^ = 12.97, *p* = 0.113; STS score: χ^2^ = 7.68, *p* = 0.466). Bootstrap calibration analysis further demonstrated smaller calibration errors for the STS Score, with lower mean absolute error (0.017 vs. 0.030) and mean squared error (0.00043 vs. 0.00405), suggesting closer agreement between predicted and observed event probabilities. These findings suggest that although STS score demonstrated superior discrimination and calibration compared with EuroSCORE II, the overall predictive performance of both models remained modest.

In logistic regression analysis, EuroSCORE II was significantly associated with PCPCs (odds ratio [OR]: 1.06; 95% CI: 1.01–1.10; *p* = 0.006). The optimal cut-off value, determined using the Youden index, was 1.63, yielding a sensitivity of 70.3% and a specificity of 42.7%. Similarly, the STS score was significantly association with PCPCs (OR: 1.05; 95% CI: 1.03–1.07; *p* < 0.001). The optimal STS score cut-off was 13.1, corresponding to a sensitivity of 52.0% and a specificity of 69.9% (Table 4). Overall, the STS score demonstrated superior discriminative performance compared with EuroSCORE II. However, both models showed only modest predictive ability, indicating limited accuracy for predicting PCPCs in older adults undergoing cardiac surgery.

Subgroup analyses according to procedure type demonstrated a consistent trend toward better discrimination of the STS score across isolated CABG, valvular surgery, and combined procedures (Table 5). However, these differences did not have statistical significance, and both models continued to demonstrate only modest predictive performance across all surgical categories.

## 4. Discussion

In this retrospective cohort study of older adults undergoing on-pump cardiac surgery, we evaluated the predictive performance of EuroSCORE II and the STS score for PCPCs. The key findings of this study are not that the STS score outperformed EuroSCORE II, but that both models demonstrated limited ability to predict postoperative cardiopulmonary complications in older adults undergoing cardiac surgery. Although the STS score showed statistically superior discrimination, the absolute predictive performance of both models remained modest (AUC 0.625 and 0.581, respectively), indicating that neither model provides sufficient accuracy for individualized morbidity prediction. These findings highlight the limitations of currently available mortality-based risk scores when applied to postoperative morbidity outcomes in elderly cardiac surgical patients.

The difference in predictive performance between the two models may be explained by their original design and intended clinical applications. EuroSCORE II, was originally developed to estimate operative mortality, focusing primary on death rather than postoperative morbidity [18,21,22]. Consequently, its ability to predict complications may be inherently limited [22,23]. Prior studies have consistently shown that mortality-focused risk models often have reduced accuracy when applied to nonfatal outcomes [22,24]. In contrast, the STS score incorporates a broader range of perioperative variables and was designed to evaluate multiple postoperative outcomes [14], explicitly including major morbidity endpoints [14,21,23,25]. This structural difference may explain the superior discrimination observed in our study.

Nevertheless, the overall predictive performance of both models remained modest. Previous studies have similarly reported limited discrimination of EuroSCORE II and STS score for postoperative morbidity, with substantially lower predictive accuracy than that observed for mortality outcomes [13,24,26]. Consistent with these findings, our study demonstrated that neither model achieved a level of discrimination sufficient for clinical meaningful individualized prediction of PCPCs. These findings suggest that traditional mortality-based risk models may not adequately capture the complex mechanisms underlying postoperative morbidity in older adults.

Prediction of PCPCs is particularly challenging because these events are multifactorial and influenced by numerous factors beyond baseline cardiovascular risk. Postoperative morbidity is affected by intraoperative physiology, perioperative management, postoperative care, and patient-specific vulnerability factors that are not fully incorporated into conventional risk models [27,28,29,30]. Furthermore, aging is associated with diminished physiological reserve, altered cardiopulmonary interactions, impaired adaptive responses, and increased susceptibility to perioperative stressors [31,32]. These age-related characteristics may further limit the performance of existing risk prediction tools in elderly cardiac surgical populations.

Several preoperative characteristics differed significantly between patients with and without complications [30,33]. Patients who developed PCPCs more frequently had congestive heart failure, higher NYHA functional class, malignancy, and preoperative anemia [34,35]. Beyond these established cardiac risk factors, our study also identified several markers of physiological vulnerability, including frailty, lower serum albumin levels, and impaired renal function. These variables may reflect diminished physiological reserve, impaired nutritional status, and reduced resilience to perioperative stress, all of which are particularly relevant in older adults undergoing cardiac surgery. Although not fully incorporated into traditional mortality-based risk models such as EuroSCORE II and STS score, these factors may contribute substantially to postoperative morbidity. Our findings support the concept that postoperative outcomes in elderly cardiac surgical patients are influenced not only by conventional cardiovascular risk factors but also by geriatric-specific characteristics, including frailty, functional status, nutritional status, and biological vulnerability.

The clinical implications of our findings require careful consideration. Although STS score demonstrated statistically superior discrimination compared with EuroSCORE II, its predictive performance remained insufficient for individualized clinical decision-making. Therefore, both EuroSCORE II and STS score should be regarded as supportive risk assessment tools rather than definitive predictors of postoperative morbidity. Future risk stratification strategies may require integration of frailty assessments, functional capacity, nutritional status, and perioperative variables to better capture the complex determinants of postoperative cardiopulmonary complications in older adults undergoing cardiac surgery.

This study has some limitations. First, its retrospective design may introduce selection bias and limit causal inference. Second, the single-center setting may reduce generalizability. Third, EuroSCORE II and the STS score are composite models with overlapping variables, raising the possibility of collinearity in direct comparisons. Fourth, the primary outcome was a composite cardiopulmonary endpoint that included complications with differing pathophysiological mechanisms and clinical significance, such as postoperative atrial fibrillation, low cardiac output syndrome, prolonged ventilation, and pneumonia. Patients who experienced one or more of these events were classified as having a PCPC, regardless of the number of individual complications. Furthermore, postoperative AF was the most frequent component of the composite endpoint, occurring in 134 patients (21%). The relatively high frequency of this event may have influenced the overall event rate and the observed predictive performance of both risk models. Although the composite endpoint increased statistical power and reflected the overall burden of postoperative cardiopulmonary morbidity, it may have obscured differences in predictive performance for individual complications. Finally, postoperative complications are influenced by perioperative management strategies, including ventilatory management, transfusion practices, hemodynamic optimization, and postoperative critical care protocols, which were not consistently available in the retrospective database and therefore could not be explicitly modeled in this study.

## 5. Conclusions

EuroSCORE II and the STS score were both associated with postoperative cardiopulmonary complications but demonstrated only modest discriminative ability. Although the STS score performed significantly better than EuroSCORE II, neither model achieved sufficient accuracy for clinically meaningful individualized risk prediction. Future risk prediction strategies should incorporate frailty and other geriatric-specific factors to improve postoperative morbidity prediction in older cardiac surgical patients.

## Figures and Tables

**Table 1:** Incidence of postoperative cardiorespiratory complications.

Postoperative Cardiorespiratory Complications	*n* = 638
Cardiac complication	198 (31.0%)
Postoperative atrial fibrillation	134 (21.0%)
Cardiac tamponade	13 (2.0%)
AICD/PPM used	15 (2.4%)
Postoperative cardiac arrest	18 (2.8%)
Postoperative ECMO	5 (0.8%)
Postoperative IABP	40 (6.3%)
Respiratory complication	157 (24.6%)
Prolong intubation	76 (11.9%)
Reintubation	27 (4.2%)
Pneumonia	41 (6.4%)
Atelectasis	41 (6.4%)
Pleural effusion	36 (5.6%)

AICD, automated implantable cardioverter defibrillator; PPM, permanent pacemaker; ECMO, extracorporeal membrane oxygenation; IABP, intra-aortic balloon pump.

**Table 2:** Baseline characteristics of patients with and without postoperative cardiopulmonary complications.

	Cardiopulmonary Complications		
Variable	No (*n* = 365)	Yes (*n* = 273)	*p*-Value
Age, median (IQR), years	68 (63–73)	69 (64–74)	0.342
Sex			0.385
Male	238 (65.2%)	168 (61.5%)	
Female	127 (34.8%)	105 (38.5%)	
Body weight (kg), median (IQR)	62 (54–70)	60 (53–67)	0.027
Body height (cm), median (IQR)	160 (155–166)	160 (153–165)	0.064
Body mass index (kg/m^2^), median (IQR)	24.2 (21.8–26.8)	23.5 (21.6–26.2)	0.162
Preoperative comorbidity	353 (96.7%)	267 (97.8%)	0.561
Ischemic heart disease	242 (66.3%)	188 (68.9%)	0.550
History of percutaneous coronary intervention	32 (9.0%)	33 (12.4%)	0.219
History of angina	242 (68.0%)	184 (68.9%)	0.872
History of congestive heart failure	106 (29.0%)	129 (47.3%)	<0.001
Transient ischemic attack	0 (0%)	2 (0.7%)	0.183
Cerebrovascular accident with deficit	15 (4.2%)	13 (4.9%)	0.845
Cerebrovascular accident without deficit	25 (6.8%)	15 (5.5%)	0.594
Hypertension	253 (71.1%)	199 (74.5%)	0.385
Diabetes mellitus	100 (28.1%)	77 (28.8%)	0.908
Insulin-dependent diabetes mellitus	20 (5.5%)	27 (9.9%)	0.050
Cancer	10 (2.8%)	23 (8.6%)	0.003
Atrial fibrillation	61 (16.7%)	54 (19.8%)	0.372
Peripheral arterial disease	6 (1.7%)	3 (1.1%)	0.739
Chronic obstructive pulmonary disease	7 (2.0%)	12 (4.5%)	0.114
Preoperative clinical profiles			
NYHA functional class III–IV	87 (23.8%)	101 (37.0%)	<0.001
Left ventricular ejection fraction (%), median (IQR)	60 (45–68.5)	59 (40–68.8)	0.094
EuroSCORE II, median (IQR)	1.8 (1.2–3)	2.3 (1.4–4)	<0.001
STS score, median (IQR)	9.8 (6.7–14.5)	13.4 (8.1–21.4)	<0.001
mFI-11			0.008
Robust (mFI-11 = 0)	39 (10.7%)	25 (9.2%)	
Prefrail (mFI-11 <= 0.27	198 (54.2%)	119 (43.6%)	
Frail (mFI-11 > 0.27)	128 (35.1%)	129 (47.3%)	
ASA classification			<0.001
3	347 (95.1%)	230 (84.2%)	
4	18 (4.9%)	61 (9.6%)	
Preoperative anemia	187 (51.2%)	174 (63.7%)	0.002
Preoperative creatinine clearance (mL/min), median (IQR)	53.8 (41.1–70.4)	47.7 (34.4–65.1)	<0.001
Preoperative albumin level available	304 (83.3%)	232 (85.0%)	0.639
Albumin (mg/dL), median (IQR)	4.1 (3.8–4.3)	4 (3.6–4.2)	<0.001
Operative characteristics			
CABG	197 (54.0%)	141 (51.6%)	0.616
Valvular surgery	119 (32.6%)	88 (32.2%)	0.990
CABG + valve surgery	49 (13.4%)	45 (16.5%)	0.334
Intraoperative variables			
Operating room time (min), median (IQR)	310 (270–375)	330 (285–390)	0.007
CPB time (min), median (IQR)	107 (81.8–139.2)	117 (84–154)	0.019
AXC time (min), median (IQR)	74 (51–101.5)	80.5 (55–110.2)	0.020
Blood loss (mL), median (IQR)	500 (500–600)	500 (500–700)	0.008
Use of blood components	363 (99.5%)	271 (99.3%)	1.000
Packed red cells (mL), median (IQR)	319 (238–505)	481 (277.5–610.5)	<0.001
Fresh frozen plasma (mL), median (IQR)	583 (534–651)	599 (551.5–998.2)	0.019
Platelet concentrate (mL), median (IQR)	207.5 (0–346.8)	303 (0–353)	0.027
Cryoprecipitate (mL), median (IQR)	0 (0–0)	0 (0–0)	0.059

IQR, interquartile range; STS, Society of Thoracic Surgeons; mFI, modified Frailty Index; ASA, American Society of Anesthesiologists; CABG, coronary artery bypass grafting; CPB, cardiopulmonary bypass; AXC, aortic cross-clamp.

**Table 3:** Discrimination, calibration, and overall predictive performance of EuroSCORE II and STS score for postoperative cardiopulmonary complications.

Model	AUC (95% CI)	Brier Score	Hosmer–Lemeshow χ^2^ (df = 8)	*p*-Value	Calibration MAE	Calibration MSE
EuroSCORE II	0.581 (0.536–0.626)	0.240	12.97	0.113	0.030	0.00405
STS Score	0.625 (0.580–0.669)	0.231	7.68	0.466	0.017	0.00043

AUC, area under the receiver operating characteristic curve; CI, confidence interval; MAE, mean absolute error; MSE, mean squared error.

**Table 4:** Predictive performance of EuroSCORE II and the STS score for postoperative cardiopulmonary complications.

Variable	OR (95% CI)	*p*-Value	AUC (95% CI)	Optimal Cut-Off	Sensitivity (%)	Specificity (%)	PPV (%)	NPV (%)
EuroSCORE II	1.06 (1.01–1.10)	0.021	0.581 (0.536–0.626)	1.63	70.3	42.7	47.9	65.8
STS Score	1.05 (1.03–1.07)	<0.001	0.625 (0.580–0.669)	13.1	52.0	69.9	56.3	66.1

EuroSCORE II, The European System for Cardiac Operative Risk Evaluation II; STS, Society of Thoracic Surgeons; OR, odds ratio; CI, confidence interval; AUC, area under the curve; PPV, positive predictive value; NPV, negative predictive value.

**Table 5:** Subgroup analysis of the predictive performance of EuroSCORE II and STS score according to procedure type.

Procedure	EuroSCORE II AUC (95% CI)	STS Score AUC (95% CI)	*p*-Value
CABG	0.56 (0.50–0.63)	0.60 (0.54–0.66)	0.086
Valve	0.61 (0.53–0.69)	0.65 (0.58–0.73)	0.235
CombineCABG + Valve	0.60 (0.48–0.71)	0.69 (0.58–0.80)	0.074

AUC, area under the receiver operating characteristic curve; CI, confidence interval; STS, Society of Thoracic Surgeons; CABG, coronary artery bypass grafting.

## Data Availability

All data generated or analyzed during this study are included in this published article.

## Abbreviations

The following abbreviations are used in this manuscript:

ASA	American Society of Anesthesiologists
AUC	Area under the curve
AXC	Aortic cross-clamp
BMI	Body mass index
CABG	Coronary artery bypass grafting
CI	Confidence interval
CPB	Cardiopulmonary bypass
EuroSCORE II	European System for Cardiac Operative Risk Evaluation II
IQR	Interquartile range
NYHA	New York Heart Association
OR	Odds ratio
PCPCs	Postoperative cardiopulmonary complications
ROC	Receiver operating characteristic
STS	Society of Thoracic Surgeons
